# The impact of tunnel mutations on enzymatic catalysis depends on the tunnel-substrate complementarity and the rate-limiting step

**DOI:** 10.1016/j.csbj.2020.03.017

**Published:** 2020-03-25

**Authors:** Piia Kokkonen, Michaela Slanska, Veronika Dockalova, Gaspar P. Pinto, Esther M. Sánchez-Carnerero, Jiri Damborsky, Petr Klán, Zbynek Prokop, David Bednar

**Affiliations:** a Loschmidt Laboratories, Department of Experimental Biology and RECETOX, Faculty of Science, Masaryk University, Brno, Czech Republic; b International Clinical Research Centre, St. Ann’s Hospital, Brno, Czech Republic; c Department of Chemistry and RECETOX, Faculty of Science, Masaryk University, Brno, Czech Republic

**Keywords:** BDP, 8-chloromethyl-3,5-dimethyl-4,4-difluoro-4-bora-3a,4a-diaza-s-indacene, COU, 4-(bromomethyl)-6,7-dimethoxycoumarin, MD, molecular dynamics, MSM, Markov state model, NAC, near-attack conformer, Enzyme kinetics, Enzyme mutation, Substrate specificity

## Abstract

Transport of ligands between bulk solvent and the buried active sites is a critical event in the catalytic cycle of many enzymes. The rational design of transport pathways is far from trivial due to the lack of knowledge about the effect of mutations on ligand transport. The main and an auxiliary tunnel of haloalkane dehalogenase LinB have been previously engineered for improved dehalogenation of 1,2-dibromoethane (DBE). The first chemical step of DBE conversion was enhanced by L177W mutation in the main tunnel, but the rate-limiting product release was slowed down because the mutation blocked the main access tunnel and hindered protein dynamics. Three additional mutations W140A + F143L + I211L opened-up the auxiliary tunnel and enhanced the product release, making this four-point variant the most efficient catalyst with DBE. Here we study the impact of these mutations on the catalysis of bulky aromatic substrates, 4-(bromomethyl)-6,7-dimethoxycoumarin (COU) and 8-chloromethyl-4,4′-difluoro-3,5-dimethyl-4-bora-3a,4a-diaza-*s*-indacene (BDP). The rate-limiting step of DBE conversion is the product release, whereas the catalysis of COU and BDP is limited by the chemical step. The catalysis of COU is mainly impaired by the mutation L177W, whereas the conversion of BDP is affected primarily by the mutations W140A + F143L + I211L. The combined computational and kinetic analyses explain the differences in activities between the enzyme-substrate pairs. The effect of tunnel mutations on catalysis depends on the rate-limiting step, the complementarity of the tunnels with the substrates and is clearly specific for each enzyme-substrate pair.

## Graphical abstract

**Figure:**
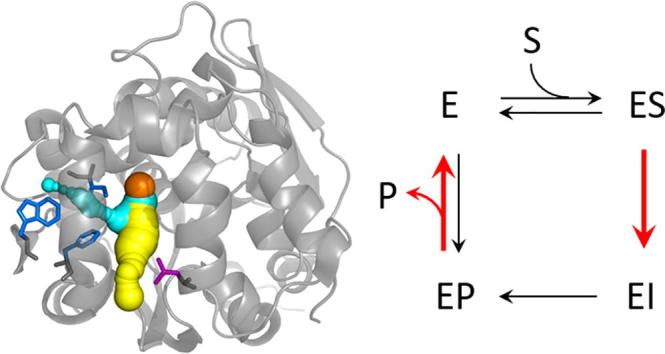

## 1 Introduction

Haloalkane dehalogenases have evolved to catalyze the cleavage of carbon-halogen bonds in various halogenated hydrocarbons [1], [2]. These compounds can be produced by bacteria, fungi, plants, and algae [3]. However, most haloalkanes are of human origins because they are widely used as flame retardants, fire extinguishers, refrigerants, propellants, solvents and pharmaceuticals [4]. Because most of these chemicals have only been introduced in the last century, it is interesting to study the molecular evolution of enzymes, such as haloalkane dehalogenases, that decontaminate these toxic compounds which continue to cause environmental issues [5], [6]. Structurally characterized haloalkane dehalogenases possess distinct access tunnels that are used for the exchange of ligand between the buried active site and surrounding solvent [2]. Therefore, haloalkane dehalogenases have become the archetype enzymes for the analysis and engineering of protein tunnels [7], [8], [9], [10], [11].

**Fig. 1:**
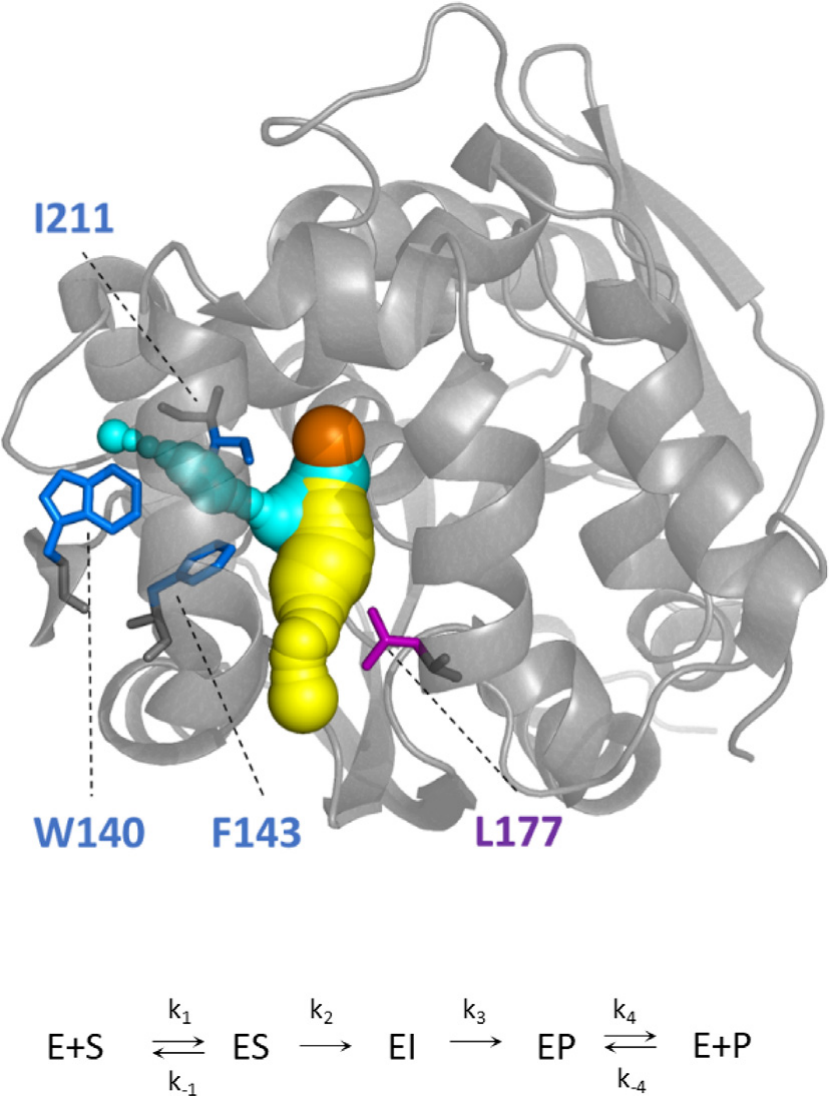
The structure of LinBwt (PDB ID 1 MJ5) with the main p1 tunnel (yellow) and the auxiliary p3 tunnel (cyan) and the kinetic scheme for the catalytic reaction. The catalytic site is depicted by the cocrystallized chloride ion (orange). The purple-labeled residue L177 is mutated in LinB32 and LinB86 as L177W. The blue-labeled residues are mutated in LinB86 as W140A, F143L and I211L. Legend: E = enzyme, S = substrate, ES = enzyme-substrate complex, EI = covalently bound alkyl-enzyme intermediate, EP = enzyme-product complex, P = product. *k*_1_ = rate constant for the substrate binding, *k*_−1_ = rate constant for the substrate release, *k*_2_ = rate constant for the S_N_2 step, *k*_3_ = rate constant for the hydrolytic step, *k*_4_ = rate constant for the product release, *k*_−4_ = rate constant for the product binding. (For interpretation of the references to color in this figure legend, the reader is referred to the web version of this article.)

One of the most studied haloalkane dehalogenases is LinB from *Sphingobium japonicum UT26* which participates in the degradation of γ-hexachlorocyclohexane, also called gamma-HCH, gamma-BHC and lindane [12], [13]. It continues to be extensively studied both with experimental and computational methods to understand its degradation mechanisms, kinetics and enantioselectivity [14], [15], [16]. During the years of studying this enzyme, we have engineered two LinB variants, namely LinB32 and LinB86 [9], [17], with interesting catalytic properties without any changes to the residues of the catalytic site (Fig. 1). LinB32 has a mutation L177W in its main tunnel which hinders the transport of substrates and products but provides stabilization of the protein conformation for the first chemical reaction step [18]. LinB86 has the same modification and three additional mutations (W140A + F143L + I211L) in the auxiliary access tunnel p3 [9]. These three mutations open an additional p3 tunnel and increase the conformational dynamics of the main tunnel to assist with the ligand transport, while L177W still contributes to the increase in the first catalytic step, making this enzyme the most efficient haloalkane dehalogenase in the degradation of 1,2-dibromoethane (DBE).

In a previous substrate screening [19], we identified a diverse set of substrates catalyzed by various dehalogenases, including LinB. Among the substrates were some fluorescent molecules which differ from the natural substrates of haloalkane dehalogenases by both their size and aromaticity. One of these molecules was 4-(bromomethyl)-6,7-dimethoxycoumarin (COU) which was well catalyzed by wild type LinB. Inspired by this to find more fluorescent substrates for the enzymes, we performed another substrate screening [20] resulting in the application of a BODIPY derivative, 8-chloromethyl-4,4′-difluoro-3,5-dimethyl-4-bora-3a,4a-diaza-*s*-indacene (BDP). Interestingly, the structure–activity relationships of the three substrates – DBE, COU and BDP – with the three LinB variants – LinBwt, LinB32 and LinB86 – are dramatically different.

In this study, we use transient kinetic experiments and molecular dynamics simulations with Markov state models (MSMs) to characterize the differences between the enzyme variants and the three substrates in detail. MSMs can be used to study the frequency of certain events (i.e., ligand binding). The Markov states are generated by calculating a certain metric (such as distance of a ligand to the active site), clustering the results and calculating two matrices: the matrix which tells in which state the system is, and transition matrix that contains the probability of the system being in this or the other states after the lag time. From these, the equilibrium probability of each state can be calculated, in addition to other kinetic parameters describing the system [21].

This study reveals that rational tunnel design must reflect the rate-limiting step and mutual complementarity between the tunnel and the ligand. The effects of mutations cannot be generalized from one substrate to another and tunnels need to be individually tailored for each enzyme-substrate pair.

## 2 Results

**Fig. 2:**
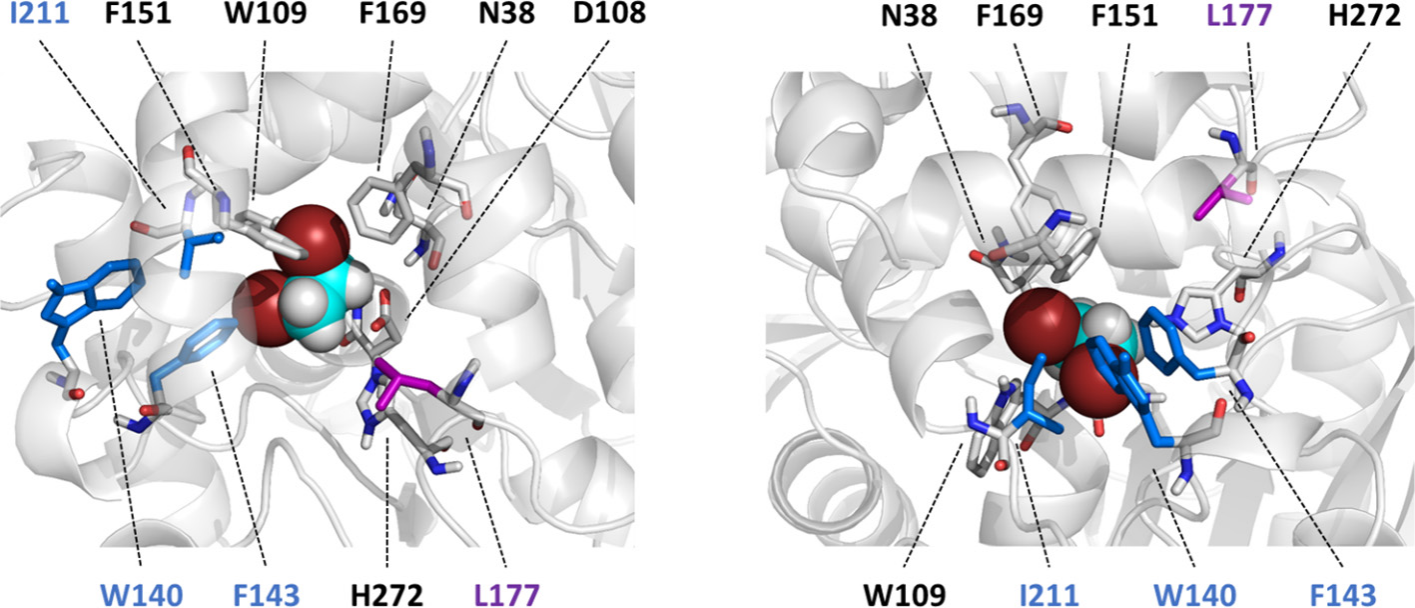
The NAC conformation of DBE in LinBwt viewed from the main tunnel (left) and from the p3 tunnel (right). As a small molecule, DBE does not interact with many residues besides the catalytic pentad D108, H272, N38, W109 and E138 (not within 4 Å to DBE and thus not shown). DBE is shown as spheres and residues within 4 Å from DBE and the mutated residues are shown as sticks. The mutated residues in the variants follow the color scheme presented in Fig. 1. DBE is shown with the following color coding: brown = bromine, cyan = carbon and white = hydrogen. (For interpretation of the references to color in this figure legend, the reader is referred to the web version of this article.)

**Fig. 3:**
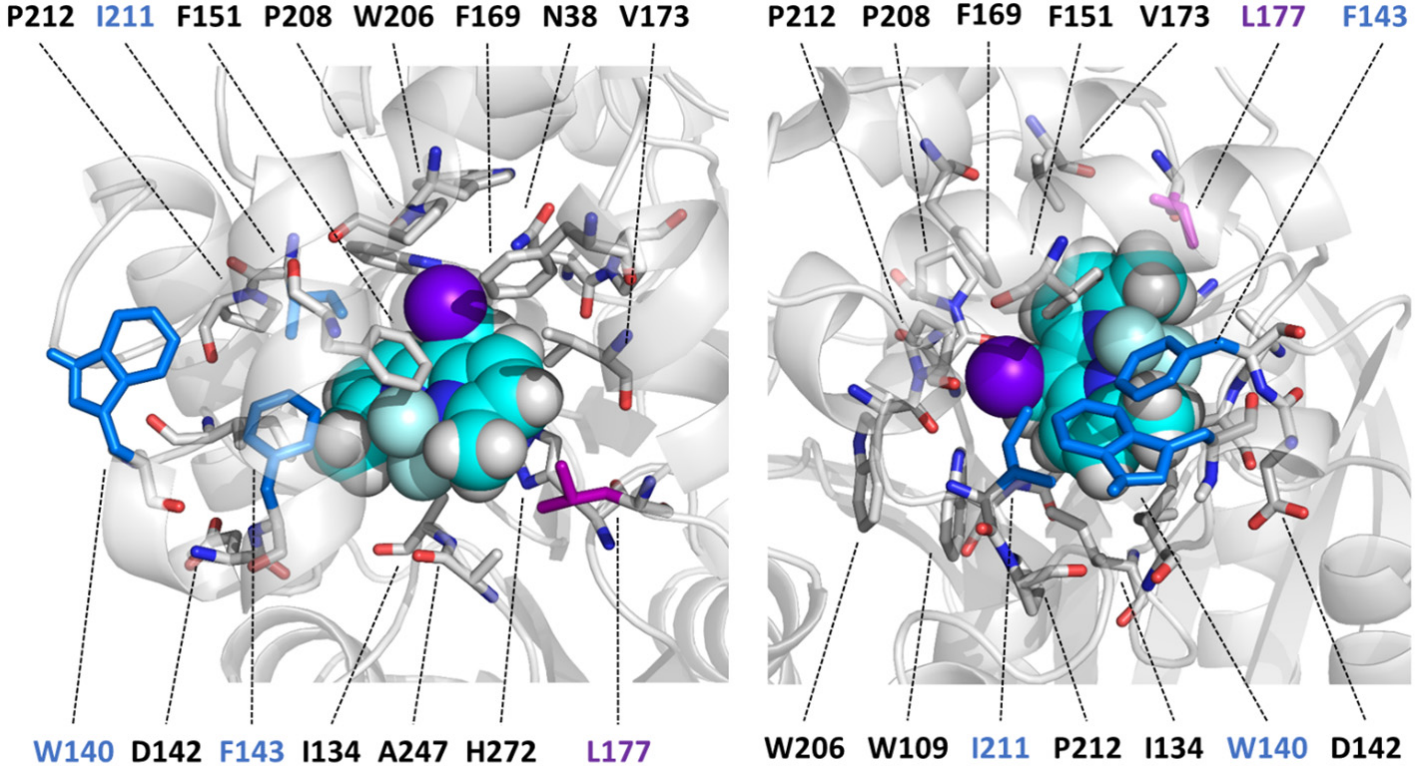
The NAC conformation of BDP in LinBwt viewed from the main tunnel (left) and from the p3 tunnel (right). The cleaved halide (chlorine) of BDP is situated in the middle of the aromatic ring system which causes parts of the molecule to bind to the p3 region while the other side fills the active site almost completely. BDP is shown as spheres, and residues within 4 Å from BDP and the in LinB32 and LinB86 mutated residues are shown as sticks following color scheme of Fig. 1. BDP is shown with the following color scheme: purple = chlorine, cyan = carbon, light cyan = fluorine, blue = nitrogen and white = hydrogen. (For interpretation of the references to color in this figure legend, the reader is referred to the web version of this article.)

**Fig. 4:**
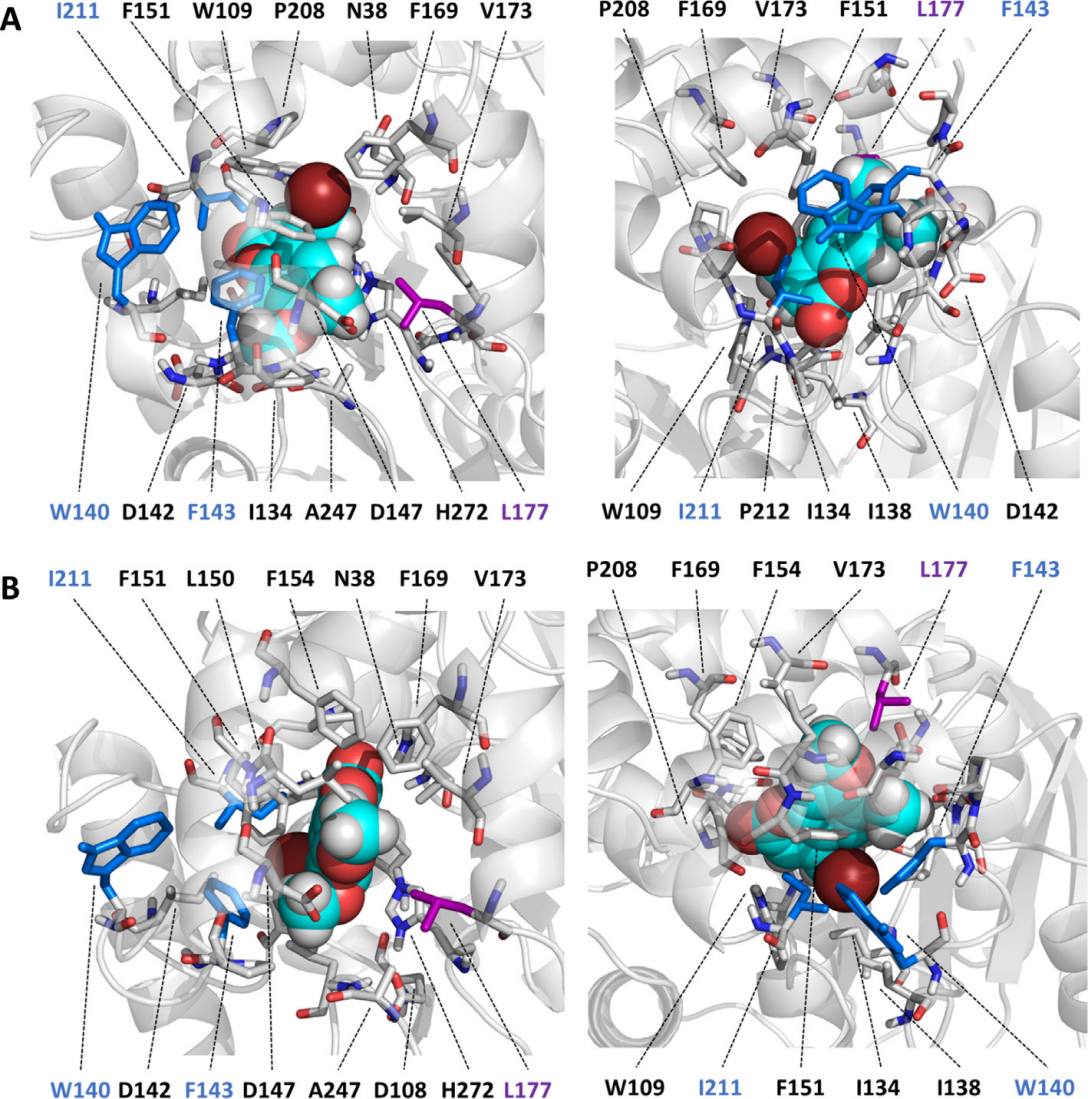
A) The NAC conformation of COU in LinBwt viewed from the main tunnel (left) and from the p3 tunnel (right). B) The tight binding conformation of COU in LinBwt viewed from the main tunnel (left) and from the p3 tunnel (right). In this binding mode, the carbonyl oxygen binds between the halide stabilizing residues N38 and W109. The cleaved halide (bromine) of COU is situated at the edge of the aromatic ring system which causes COU to mostly bind to p1 region in both binding modes. COU is shown as spheres, and residues within 4 Å from COU and the in LinB32 and LinB86 mutated residues are shown as sticks following color scheme of Fig. 1. COU is shown with the following color scheme: brown = chlorine, cyan = carbon, red = oxygen and white = hydrogen. (For interpretation of the references to color in this figure legend, the reader is referred to the web version of this article.)

The kinetic data of three protein variants LinBwt, LinB32 and LinB86 with the substrates DBE, BDP and COU are shown in Table 1. The reactive binding mode, sometimes called the near-attack conformer (NAC) of all substrates observed in the simulations with the LinBwt, is shown in Fig. 2, Fig. 3, Fig. 4. Below we discuss different kinetic parameters and the corresponding populations of bound/unbound states for the individual substrate molecules.

**Table 1:** The steady-state and pre-steady state kinetics of DBE, BDP and COU reaction with LinB variants. Individual rate constants (±standard errors) obtained from a global fit of the kinetic data to the model of the catalytic cycle (Fig. 1). The rate-limiting steps of the catalytic cycle are highlighted in bold. Statistical significance of difference between kinetic values (LinB32 compared to wild type, LinB86 compared to LinB32) indicated at *p-value <0.05 and **p-value <0.01.

			LinBwt	LinB32	LinB86
DBE	*Steady-state kinetics*				
	*k*_cat_	s^−1^	12 ± 4	3.1 ± 0.1*	57 ± 3**
	*K*_m_	μM	1 700 ± 200	420 ± 30**	2 350 ± 30**
	*k*_cat_/*K*_m_	μM^−1^ s^−1^	0.007 ± 0.002	0.007 ± 0.001	0.024 ± 0.001**
	*Pre-steady-state kinetics*				
	*K*_S_	μM	21 ± 1 × 10^3^	37 ± 2 × 10^3^**	17 ± 1 × 10^3^**
	*k*_2_	s^−1^	120 ± 10	330 ± 10**	350 ± 10
	*k*_3_	s^−1^	139 ± 5	109 ± 4**	340 ± 10**
	*k*_4_	s^−1^	**10 ± 1**	3.2 ± 0.1**	70 ± 5**

**BDP**	*Steady-state kinetics*				
	*k*_cat_	s^−1^	2.3 ± 0.2	1.1 ± 0.1**	0.015 ± 0.001**
	*K*_m_	μM	17 ± 1	24 ± 4*	26 ± 2
	*k*_cat_/*K*_m_	μM^−1^ s^−1^	0.14 ± 0.01	0.046 ± 0.009**	0.0006 ± 0.0001**
	*Pre-steady-state kinetics*				
	*K*_S_	μM	38 ± 2	23 ± 1**	70 ± 10**
	*k*_1_	μM^−1^ s^−1^	2.4 ± 0.1	2.8 ± 0.1**	0.024 ± 0.002**
	*k*_−1_	s^−1^	90 ± 3	65 ± 1**	1.7 ± 0.3**
	*k*_2_	s^−1^	**5.9 ± 0.2**	2.1 ± 0.1**	0.18 ± 0.01**
	*k*_3_	s^−1^	>10	12 ± 1	0.011 ± 0.001**
	*k*_4_	s^−1^	>10	>10	18 ± 3

**COU**	*Steady-state kinetics*				
	*k*_cat_	s^−1^	3.9 ± 0.2	0.68 ± 0.02**	0.89 ± 0.03**
	*K*_m_	μM	0.7 ± 0.5	13 ± 1**	30 ± 2**
	*k*_cat_/*K*_m_	μM^−1^ s^−1^	6 ± 4	0.052 ± 0.004**	0.030 ± 0.002**
	*Pre-steady-state kinetics*				
	*K*_S_	μM	<1	80 ± 9	240 ± 30**
	*k*_1_	μM^−1^ s^−1^	5.1 ± 0.6	0.50 ± 0.05**	0.10 ± 0.01**
	*k*_−1_	s^−1^	–a	40 ± 2	24 ± 2**
	*k*_2_	s^−1^	**6.5 ± 0.1**	3.1 ± 0.2**	3.6 ± 0.2*
	*k*_3_	s^−1^	18 ± 1	1.14 ± 0.03**	1.4 ± 0.1**

a The reverse rate could not be obtained by the global fit of kinetic data (*k*_−1_ ≪ *k*_2_). The data for DBE conversion were determined at 37 °C in a glycine buffer pH 8.6 [17]. The kinetic data for BDP conversion by LinBwt [20], LinB32 (Fig. S10) and LinB86 (Fig. S11), and the kinetic data for conversion of COU by LinB variants [20], [42] were recorded at 30 °C in phosphate buffer pH 8.0 with 10% DMSO. In the case of COU, the similar value of quantum yield for the alkyl-enzyme intermediate and product did not allow distinction between the last kinetic steps (i.e., hydrolysis and product release). A simplified three-step model including substrate binding, cleavage of carbon-halogen bond and last step leading to free enzyme was applied for fitting the reaction of LinB variants with COU (four-step step model was not supported by the obtained data). The equilibrium constant for dissociation of the enzyme-substrate complex for BDP and COU was calculated from individual rate constants *k*_1_ and *k*_−1_ obtained from global fitting (*K*_s_ = *k*_−1_/*k*_1_). In a case of DBE, the *K*_S_ was derived as the only parameter from global fit since substrate-binding reached rapid-equilibria and individual kinetic constant for substrate binding and dissociation, *k*_1_ and *k*_−1_, could not be obtained from the data.

### 2.1 1,2-Dibromoethane (DBE)

The catalytic cycle of a small, polar and aliphatic molecule DBE is limited by the product release (Table 1). As a small substrate, DBE passes the access tunnel of LinBwt smoothly and reaches the reactive binding mode without conformational changes required from the enzyme. DBE gets converted efficiently by S_N_2 nucleophilic substitution and hydrolysis steps into the products: 2-bromoethanol and bromide ion. In our previous studies, we have shown that the product release is the rate-limiting step of DBE catalysis for the studied LinB variants [9], [17].

Because DBE is a small molecule, it does not interact directly with other than the catalytic pentad of the active site in the NAC (Fig. 2). It is also important to note that all the mutations in LinB32 and LinB86 are too far to directly interact with DBE when it is bound in NAC. It is therefore not surprising that, in the case of DBE, the mutations in LinB32 and LinB86 mostly affect the transport of ligands in and out from the active site.

The *k*_cat_ of LinBwt, LinB32 and LinB86 with DBE is controlled by the rate-limiting product release *k*_4_. By hindering the product release with the bulky L177W mutation, the catalytic rate and the product release slowed down in LinB32, even though the conversion rate increased, particularly for the S_N_2 reaction (Table 1). The MSMs obtained from the molecular dynamics simulations display a clear NAC state in LinB32 and LinB86, but not in LinBwt (Supporting Information Figs. S1–S4). However, there are multiple snapshots in LinBwt simulations, where DBE is bound in the NAC conformation within the “bound” Markov state. This data supports the experimental observation that L177W mutation enhances the first catalytic step by stabilizing the NAC by sterically decreasing the size of the active site and constraining the dynamics of the enzyme [17].

With LinB86, the extra mutations in p3 area introduce new opening, faster dynamics and increased transfer of water, substrates and products to and from the active site [9], [17]. This all comes in addition to retaining the stabilized NAC conformation by the L177W mutation, making LinB86 the most efficient DBE catalyst of the three tested enzymes (*k*_cat_ = 57 ± 3 s^−1^; *K*_m_ = 2350 ± 30 μM). Surprisingly, even though the mutations introduced to p3 opened this tunnel for ligand transport, the main tunnel remains the main route for substrate binding and product release. The fluxes from one Markov state to another reveal that the binding of DBE happens through the main tunnel 75% of the time and through p3 25% of the time (Supporting Information Table S1 and Fig. S5). This is in agreement with the previous simulations where the transport of products through p3 was observed in ~10% of the cases [9]. The discrepancy between increased product release and the opened p3 tunnel which is not so frequently used is explained by an increase in dynamical movements of p1 tunnel. The smaller residues in p3 tunnel allow the movement of the α5 helix in such a way that p1 tunnel can adopt both open and closed conformations. In the open state, the tunnel reaches >18 Å diameter, which explains the increase in product release, even when the tunnel blocking residue L177W is still present [17].

### 2.2 8-Chloromethyl-4,4′-difluoro-3,5-dimethyl-4-bora-3a,4a-diaza-*s*-indacene (BDP)

The rate of bulky and hydrophobic BDP binding to the active site of LinBwt (*k*_1_) is significantly slower in comparison with small and polar DBE. However, the equilibrium binding affinity (*K*_S_ = *k*_−1_/*k*_1_) is in the micromolar range and thus significantly more effective than the binding of DBE, possessing equilibrium constant at the millimolar range (Table 1). This strong substrate affinity of bulky BDP is associated with a slow velocity of following catalytic step (*k*_2_), the S_N_2 nucleophilic substitution, which is the rate-limiting for LinBwt and LinB32. While the introduction of L177W in p1 did not affect significantly kinetics of BDP conversion, mutations W140A + F143L + I211L in p3 of LinB86 variant induced dramatic changes in enzyme kinetics. Both the rate of substrate binding and the S_N_2 reaction step slowed down considerably. The largest impact was observed also on the second catalytic step (*k*_3_), hydrolysis of alkyl-enzyme intermediate, which became rate determining in the reaction of LinB86 with BDP.

By restricting the size and the dynamics of the main tunnel in LinB32, the binding of BDP to productive configuration turns slightly more difficult. The slightly reduced catalytic activity of LinB32 (*k*_cat_ decreased from 2.3 to 1.1 s^−1^) correlates with a decrease in the rate constant for nucleophilic substitution S_N_2 from 5.9 to 2.1 s^−1^. The introduced L177W mutation constrains the main access tunnel and this residue needs to adopt an unfavored conformation for BDP to achieve the NAC in the active site. The NAC of BDP was only observed as a clear Markov state in LinBwt simulations (Supporting Information Fig. S2).

The largest impact on the catalytic activity towards BDP was observed for the mutant LinB86 with the turnover number (*k*_cat_) decreased to 0.015 s^−1^. The transient kinetic data showed a significant decrease in the rate of substrate binding as well as the rate of both chemical steps. The rate limitation of the reaction shifted from the S_N_2 step (*k*_2_) observed for LinBwt and LinB32 to the hydrolytic step (*k*_3_) for LinB86. This can be accounted for by a steric blockage of L177W mutation, as well as the lost interactions around the p3 tunnel. BDP is too big to pass through p3 tunnel but the molecule interacts with this region of the enzyme in the NAC (Fig. 3). By mutating F143L, the critical aromatic π-π stacking interaction with BDP is lost, whereas the other two mutations, I211L and W140A, further decrease the interaction possibilities at this site and thus destabilize the NAC, which contributes to the loss of catalytic activity.

### 2.3 4-(Bromomethyl)-6,7-dimethoxycoumarin (COU)

Similarly to BDP, bulky and hydrophobic COU binds slowly and tightly, particularly when compared to small and polar DBE substrate. The binding occurs through the main tunnel because the molecule is too big to penetrate through the p3 tunnel, even in LinB86, where the mutations make the tunnel larger. Interestingly, the affinity (*K*_S_) of LinBwt for COU was even stronger compared to BDP. Still, LinBwt kept uncompromised rates of following steps in the catalysis of COU. In fact, it is the most efficiently processed substrate found for LinBwt or any other wild type haloalkane dehalogenase to date. Similarly to BDP, the rate-limiting step of COU catalysis is the S_N_2 step in LinBwt. In contrast to BDP, the reaction with COU was strongly affected by the first substitution L177W (LinB32), and this effect was not significantly altered by additional mutations W140A + F143L + I211L in p3 leading to LinB86 variant. The turnover number (*k*_cat_) was reduced from 3.3 s^−1^ observed for LinBwt to 0.7 and 0.9 s^−1^ obtained for LinB32 and LinB86, respectively. Interestingly, the single mutation L177W shifted the rate-limiting step in the reaction of COU from nucleophilic substitution (*k*_2_) to the last catalytic step (*k*_3_) for both, LinB32 and LinB86. The transient kinetic data obtained with COU provided information only for simplified three steps kinetic scheme, where *k*_3_ is the step leading to the formation of free enzyme, and the rate constant includes the contribution of both the hydrolysis of alkyl-enzyme intermediate and product release [20].

The different kinetic effects observed for BDP and COU are consistent with observations provided by molecular modeling. COU binds to the NAC in LinBwt in a different way than BDP (Fig. 4A). The halide is placed at the edge of the aromatic ring system rather than in the middle which makes COU position itself along the main tunnel instead of pushing into the region of the p3 mutations. The introduction of L177W mutation disturbs this binding by forcing the protein into a more open conformation to accommodate both the ligand and the W177 in the main tunnel. W177 can also interact with COU so it does not bind deep enough into the active site to form the NAC. Since COU is not directly interacting with the residues in p3 tunnel, the effect of these mutations in LinB86 is significantly smaller than with BDP. COU has a stacking interaction with F143 in the NAC with the LinBwt, and this interaction is lost in LinB86, contributing to a lower turnover of the S_N_2 nucleophilic substitution because of destabilized NAC.

In the initial set of 25 µs of adaptive sampling simulations of COU binding, no clear NAC state could be observed for any of the enzyme variants. Strikingly, there was a tight binding mode observed, where COU binds between the halide stabilizing residues N38 and W109 with its carbonyl oxygen (Fig. 4B). This conformation had a 6% equilibrium probability representing ~33% of the bound states (Supporting Information Fig. S3). To study the occurrence of this binding mode more closely, we conducted a second set of simulations with LinBwt, where COU was initially docked into the NAC using AutoDock Vina [22]. COU could not be docked into NAC in the other variants due to steric constraints caused by L177W mutation. These simulations showed that the NAC obtained by docking is a rare state (0.7% equilibrium probability), whereas the tightly bound state still comes up to an even larger percentage (~40%) of all the states bound to the inside the protein (NAC, tightly bound and bound) (Supporting Information Fig. S4). We propose that this tight binding mode could be catalytically productive because the distances and angles of the halide and the nucleophile match those defined for the NAC (the nucleophile—C contact distance of ≤3.41 Å and the nucleophile—C—halide angle of 157–180°) in previous studies [23]. This binding mode or state is not observed in the variants which could explain the drop in catalytic activity in LinB32 and LinB86. Instead of reaching the bottom of the active site, COU tends to interact with L177W in these variants, and this residue also sterically hinders the binding of COU into the tight-binding conformation observed in LinBwt. The tightly bound state can be catalytically active, but since the halide is not stabilized by the halide stabilizing residues, the reaction likely has a higher energy barrier to proceed than in the “traditional” NAC. The higher energy barrier is compensated by the frequent occurrence of this state and the stability of this binding mode, somewhat explaining the extra-ordinarily high activity of LinBwt towards COU.

## 3 Discussion

The catalytic cycles of bulky, hydrophobic and aromatic BDP and COU molecules are limited by the chemical reaction steps, unlike the catalytic cycle of small and hydrophobic DBE which is limited by the product release. Strikingly, the binding affinity of these substrates is three orders of magnitude higher than that of DBE. However, both bulky BDP and COU ligands show slow binding kinetics. Proper positioning of the molecules in the active site is critical for the chemical reaction to proceed, and the larger molecules have less conformational possibilities at the constrained active site. Engineering of the main p1 and the auxiliary p3 tunnels has an adverse effect on the catalysis of the large substrates both in the substrate binding and the chemical reaction. In general, the larger substrates do not bind effectively to the NAC in LinB variants with engineered access pathways optimized for catalysis of significantly small aliphatic or cyclic haloalkanes [24], [25].

Newly opened auxiliary p3 tunnel is being used for access of small substrate DBE or release of products, whereas the significantly larger molecules BDP and COU cannot use this newly created tunnel for binding to the enzyme active site [9]. The main tunnel blocked by the bulky W177 in LinB32 and LinB86 remains the only entry point to the active site for BDP and COU. The bulky substrates bind with an extremely high affinity through the main tunnel but are converted poorly due to increased interactions with the mutated W177 and limited accessibility into the NAC.

Effect of tunnel engineering is strictly ligand-specific [26], and the engineering of the p3 tunnel accompanied by an increase in the protein dynamics makes LinB86 the most efficient catalyst with DBE, whereas the tunnel engineering has an adverse effect on catalysis with BDP and COU. L177W mutation makes the active site of LinB32 smaller [18], thus driving DBE to adopt the NAC more often. The mutation also constrains the access pathway and disturbs enzyme dynamics, which prevents efficient transport of substrates, water and products to and from the active site. In LinB86, this issue is solved by increasing the throughput of an additional tunnel which also re-established the dynamical behavior of the enzyme and efficient ligand transport [9], [17].

For the catalysis of BDP, the mutation L177W in LinB32 does not induce such a significant drop as observed with DBE. This residue needs to adopt a specific conformation to allow BDP binding into NAC, which explains the lowered activity. On the other hand, the mutations W140A + F143L + I211L in p3 of LinB86 are detrimental to the catalysis of BDP. By removing most of the intricate aromatic interaction network stabilizing BDP in NAC (Fig. 3), the probability of the dehalogenation reaction diminishes.

The catalytic efficiency (*k*_cat_/*K*_m_) of LinBwt with COU is two orders of magnitude higher than ever observed with any conventional substrate. Surprisingly, it was difficult to reproduce NAC of COU with any of the LinB variants in our simulations. Even by forcing the substrate into NAC through the docking in LinBwt, the state remains less common than the NAC states with the other two studied substrates. The simulations showed a tight binding of COU to LinBwt which was relatively common in both simulation sets, reflecting extremely low *K*_m_ = 1.1 μM. This binding mode has optimal distances and angles for the nucleophilic attack, and even when the halide ion is not stabilized, it could enable the catalysis, though likely with a higher energy barrier. The higher energy barrier would be compensated by the more frequent occurrence of this state, and this “additional binding mode” could explain the high specific activity of LinBwt towards COU. The mutation L177W affects the catalysis by displacing COU from the tight-binding mode and by disturbing the water transport to the active site. The mutations in p3 region in LinB86 do not have a significant effect on the catalysis of COU, since COU does not interact with them as much as BDP.

Engineering of access tunnels in the studied LinB variants was initially done to optimize the catalysis of small and polar DBE [9]. This study revealed that DBE catalysis is limited by the product release, whereas the catalysis of the aromatic and large BDP and COU is limited by the chemical steps. By intuition, one would expect the catalytic activity with the larger molecules to diminish if the main tunnel is blocked by a tryptophan moiety. This is indeed the case with LinB32. However, in the case of LinB86, increased dynamics and additional space at the active site would intuitively increase the catalytic efficiency since the larger molecules would have more space to adopt NAC. Our results show that the modifications in LinB86 are detrimental for the catalysis of either of these substrates, more so for BDP which has more interactions at the p3 region. This observation makes the application of tunnel engineering strategy more suitable for directed evolution than to rational design [11]. It is currently very easy to predict residues forming the tunnel bottleneck that can be modified by saturation or other combinatorial mutagenesis methods, while the design of optimal tunnels for a specific protein–ligand pair is extremely challenging. The results presented in this study provide important insights into the factors impacting the effects of engineered tunnels on enzymatic catalysis.

## 4 Conclusions

Engineering protein tunnels have become a generally accepted and broadly used strategy for the modification of activity, specificity, enantioselectivity and stability of enzymes. Here we investigated the structural basis and limitations of this concept by studying the effect of tunnel engineering on a diverse set of nine enzyme-ligand pairs. We applied transient kinetic experiments for dissection of all physical and chemical steps in the catalytic cycle of wild type and engineered LinB. Systematically, we studied the effects of tunnel mutations on the rates of individual steps. Moreover, we used the molecular modeling to understand the effect of the mutations on the binding of substrates to the enzyme active sites and the formation of a reactive complex. We conclude that the changes made in the access tunnels of enzymes cannot be generalized between different enzyme-substrate pairs. The effect of mutations needs to be studied for each enzyme-substrate pair separately, paying close attention to the rate-limiting step and the complementarity of the substrate to the active site and the access tunnels. The future research should, therefore, focus on the development of computational workflows to identify the tunnels most likely used for the transport of substrates. Computational approaches are needed for the rational design of tunnels that enable the efficient binding of substrates to the buried active sites and the fast release of products. Moreover, the introduction of access tunnels to compact protein structures is far from trivial because tightly packed protein cores are thermodynamically preferred and the formation of “holes” or cavities leads to a significant loss of stabilities. This can potentially be addressed by introducing dynamical gates that allow transition of tunnels from open to closed states. More research is needed for understanding the structural basis of tunnels and gates, and for grasping their fundamental design principles.

## 5 Experimental procedures

### 5.1 Materials and methods

The reagents and solvents of the highest purity available were used as purchased, or they were purified/dried using the standard methods when necessary. All glassware was oven-dried prior to use. Purification procedures were performed using silica gel columns. Flash column chromatography was performed using silica gel Merck 60 (230–400 mesh). ^1^H and ^13^C NMR spectra were obtained in CDCl_3_ on 75 and 300 MHz spectrometers. ^1^H and ^13^C chemical shifts are reported in ppm relative to the CDCl_3_ signal as an internal standard. The enzymes were produced and purified as described previously [17].

### 5.2 Synthesis of BDP

The protocol of BDP synthesis described by Dockalova et al. [20] was used. A solution of chloroacetyl chloride (0.250 mL, 3.14 mmol, 1 equiv.) in dry dichloromethane (1 mL) was added dropwise to a solution of 2-methylpyrrole (0.550 mL, 6.28 mmol, 2 equiv.) in dry dichloromethane (15 mL) under a nitrogen atmosphere at 0 °C for 30 min. The reaction mixture was stirred at room temperature for 12 h. Then, triethylamine (1.1 mL, 7.85 mmol, 2.5 equiv.) and BF_3_∙Et_2_O in diethyl ether (46%, 2 mL, 15.7 mmol, 5 equiv.) were added at 0 °C, and the resulting mixture was stirred at room temperature for 3 h. Aq. HCl (10%, 10 mL) was added, and the crude mixture was extracted with dichloromethane (3 × 10 mL). The collected organic layers were dried over anhydrous magnesium sulfate, filtered and concentrated to dryness under reduced pressure. The product was purified by flash chromatography on silica gel (hexane/dichloromethane, 6:4). Yield 200 mg (24%). Red solid. ^1^H NMR (300 MHz, CDCl_3_): *δ* (ppm) 7.17 (d, *J* = 4.2 Hz, 2H), 6.33 (d, *J* = 4.2 Hz, 2H), 4.62 (s, 2H), 2.63 (s, 6H). ^13^C NMR (75 MHZ, CDCl_3_): *δ* (ppm) 159.2, 135.7, 134.1, 127.1, 120.0, 37.5, 15.1.

### 5.3 Steady-state kinetics

The reaction of LinB variants with BDP results in an increase in fluorescence intensity due to the formation of a highly fluorescent product. Steady-state data were obtained by recording initial phases of conversion of 1–20 μM BDP by 0.2 μM LinB86 and 0.5–10 μM BDP by 0.14 μM LinB32 in microtiter plates at 30 °C in phosphate buffer (pH 8.0) with 10% DMSO by using spectrofluorometer Synergy H4 equipped with a xenon flash lamp. The excitation/emission monochromators were set to wavelengths 500/530 nm and used to measure the fluorescence intensity from the top. 20 μl of the substrate solution was added by the automatic syringe pump to the final volume of the reaction mixture of 200 μl. The microtiter plate was shaken for 2 s and then the increase in fluorescence intensity was monitored at regular time intervals. Abiotic control was also measured to deduct spontaneous hydrolysis of the substrate. Each experiment took place in 8 wells and was repeated with 3 different aliquots of the substrate.

### 5.4 Pre-steady state kinetics

Pre-steady state kinetic experiments were performed using the stopped-flow instrument SFM-300 combined with the spectrometer system MOS-500 (BioLogic, France) at 30 °C in phosphate buffer (pH 8.0) with 10% DMSO. The reaction was initiated by the rapid mixing of the substrate with the enzyme in the ratio 1:1 to the total volume of 150 μl, the total flow rate was 16 mL/s. The mixture in the reaction cuvette was continuously excited by Xe (Hg) lamp using a wavelength with 2 nm bandwidth selected by a monochromator (500 nm for BDP or 280 nm for tryptophan excitation). The long-pass filter 530 nm was employed for the detection of emitted light. The reaction was monitored by measuring the change of voltage on the photomultiplier tube corresponding to the change of fluorescence intensity accompanying the reaction. The measurement started immediately after mixing with the dead time of 1 ms. The final kinetic traces were an average of six to ten repetitions. In the case of LinB86 with BDP, for which the reaction was very slow, the kinetic data were collected using spectrofluorometer Synergy H4 in order to avoid the fast, spontaneous hydrolysis caused by continuous illumination of the sample. The single turnover was performed by mixing BDP ranging between 2 and 10 μM with 30 μM LinB86 or 5 μM BDP with 10 μM LinB32. The reaction was also investigated at fixed concentrations of BDP (10 μM) and variable concentration of LinB86 ranging between 20 and 50 μM. The concentration dependence was also analyzed by mixing 0.65 μM LinB32 with different concentrations of BDP (0.5–5 μM).

### 5.5 Data analysis and statistics

All steady-state and transient-state kinetic data were fit globally with the Global Kinetic Explorer (KinTek Corporation). Global Kinetic Explorer allowed input of a mechanism from an input model (Fig. 1) through a simple text description, and the program then derived the differential equations needed for numerical integration automatically [27]. Numerical integration of rate equations searching a set of kinetic parameters that produce a minimum χ2 value was performed using the Bulirsch–Stoer algorithm with adaptive step size, and nonlinear regression to fit data was based on the Levenberg–Marquardt method [28], [29]. The initial values of concentration for enzymes and substrates from experimental conditions are detailed in the Supplementary Information. To account for variations in the data, enzyme or substrate concentrations were slightly adjusted (±10%) to derive best fits. Residuals were normalized by sigma value for each data point. The standard error (S.E.) was calculated from the covariance matrix during nonlinear regression. In addition to S.E. values, more rigorous analysis of the variation of the kinetic parameters was accomplished by confidence contour analysis by using FitSpace Explorer (KinTek, USA). In this analysis, the lower and upper limits for each parameter were derived (Supporting Information Table S1) from the confidence contours for χ^2^ threshold at boundary 0.98 [30]. The standard error estimates in fitted parameters were propagated to yield error estimates in calculated values, the equilibrium binding constant *K*_s_ = *k*_−1_/*k*_1_ and the specificity constant *k*_cat_/*K*_m_. Values of experimental kinetic constants for constructed variants were tested for similarity by using Welch's unequal variances *t*-test. Statistical significance of kinetic values obtained after introducing mutations (LinB32 compared to wild type, LinB86 compared to LinB32) was indicated at p-value <0.05 and p-value <0.01.

The fluorescence signal (*V*) of BDP recorded by stopped-flow was defined as the sum of fluorescence intensities from all contributing species with scaling factors for each distinct species where *f* scales the fluorescence signal to the concentration of substrate. The signal $V_{1}$ measured at 500 nm excitation was defined by an Eq. (2) with two scaling factors *a* and *b* reflecting the change of fluorescence intensity corresponding to the formation of reaction intermediate (EI) and product, respectively.

(2) $$V_{1}=f_{1}\left(S+ES+a\cdot EI+b\cdot \left(EP+P\right)\right)$$

The signal $V_{2}$ measured at 280 nm excitation was defined by an Equation (3) with four scaling factors

(3) $$V_{2}=f_{2}\left(S+o\cdot ES+p\cdot EI+q\cdot EP+r\cdot P\right)$$

where *o* and *r* primarily reflect the change in FRET efficiency between BDP and tryptophan in the active site corresponding to the substrate binding and product release, respectively, and factors *p* and *q* represent the combination of FRET and change in fluorescence intensity of BDP *a* and *b* in Equation (2). Absorption and emission spectra of BDP and tryptophan and the region of donor to acceptor energy transfer (FRET) is illustrated in Supporting Fig. S12.

### 5.6 Molecular dynamics simulations

#### 5.6.1 System preparation

The crystal structures (PDB # 1MJ5 (LinBwt), PDB # 4WDQ (LinB32) and PDB # 5LKA (LinB86)) were downloaded from RSCB Protein Data Bank [31], [32], [33]. Extra ligands and ions were removed. The hydrogen atoms were added to the structures with H++ web server at pH 7.5 [34]. The water molecules from the crystal structures, which did not overlap with the protonated structures, were retained. One ligand molecule was manually set to around the entrance of the main tunnel of the enzymes using PyMol [35]. For the docked COU simulation with LinBwt, COU was docked to the catalytic site using Autodock Vina with the exhaustiveness set to 12 and the nucleophilic Asp108 used as the center of the 40 * 40 * 40 Å^3^ docking grid [22]. The calculated binding energy was −6.1 kcal mol^−1^.

The systems were solvated in a cubical water box of OPC3 (BDP) or TIP3P (DBE and COU) water molecules so that all atoms were at least 10 Å from the surface of the box using AmberTools17 [36], [37]. Cl^−^ and Na^+^ ions were added to neutralize the charge of the protein and to get a final concentration of 0.1 M. The systems were prepared with Amber-FF14SB force field [38]. The partial charges and parameters for DBE and COU using GAFF2 force field were determined using the Parameterize function of High-Throughput Molecular Dynamics (HTMD) [36]. Parameters for the boron atom in BDP were built using the bonded model approach [39]. The parameters and partial charges of BDP were calculated using Gaussian 09 E.01 [40].

#### 5.6.2 Equilibration simulations

The DBE and COU systems were equilibrated for 5 ns using the default settings of HTMD Equilibration_v2 protocol [36]. The BDP systems were first equilibrated for 500 fs NPT equilibration using Langevin thermostat with all hydrogen-containing bonds considered flexible (rigid bonds off), timestep set to a conservative value of 0.001 fs, with 1 kcal mol^−1^ Å^−2^ constraints on all heavy atoms of the protein. Then the equilibration continued with 500 fs of NPT equilibration with the Langevin thermostat without constraints. Finally, the BDP systems were equilibrated 5 ns using the second step of Equilibration_v2 protocol of HTMD (NPT equilibration with the Langevin thermostat without constraints) [36].

#### 5.6.3 Adaptive sampling

The production simulations were run as adaptive epochs of 10 * 50 ns using the Production_v6 protocol of HTMD. The metric used in the adaptive sampling was the distance of the halide atom(s) of the ligand to the N_ε_ of the halide stabilizing tryptophan. The total simulation time with DBE was 16,050 ns, 12,000 ns and 12,350 ns for LinBwt, LinB32, and LinB86, respectively. The total simulation time with BDP was 12,500 ns, 12,600 ns and 11,700 ns for LinBwt, LinB32, and LinB86, respectively. The total simulation time for COU was 25,000 ns for all the systems and 18,400 ns for the docked COU with LinBwt.

#### 5.6.4 Markov state models

The Markov state models were built by calculating a binary contact map (distance <8 Å was considered a contact) of the halide atom(s) and their adjoined carbons (the targets of the nucleophilic attack) with the Cα atoms of the protein. 5-dimensional time-lagged independent component analysis TICA was used to find the correlations of states in time with a 5 ns lag time [41]. The data were clustered using MiniBatchKmeans algorithm to 200 clusters. The implied timescale plots (Supporting Information Fig. S6), the Chapman-Kolmogorov tests (Supporting Information Figs. S7–S9) and figures of the different Markov states (Supporting Information Figs. S1–S4) of all the build models are shown in the Supporting Information.

## CRediT authorship contribution statement

**Piia Kokkonen:** Methodology, Investigation, Writing - review & editing, Visualization. **Michaela Slanska:** Investigation, Validation. **Veronika Dockalova:** Investigation, Validation. **Gaspar P. Pinto:** Investigation, Validation. **Esther M. Sánchez-Carnerero:** Investigation, Validation. **Jiri Damborsky:** Conceptualization, Supervision, Writing - review & editing, Funding acquisition. **Petr Klán:** Conceptualization, Supervision. **Zbynek Prokop:** Conceptualization, Supervision, Funding acquisition, Writing - review & editing, Visualization. **David Bednar:** Conceptualization, Supervision, Writing - review & editing.
