# Supplementary material for: The impact of tunnel mutations on enzymatic catalysis depends on the tunnel-substrate complementarity and the rate-limiting step
Source: Comput Struct Biotechnol J. 2020 Mar 25;18:805–13. doi: 10.1016/j.csbj.2020.03.017 (PMC7152659; doi:10.1016/j.csbj.2020.03.017)
Supplement: Supplementary data 1 [file mmc1.docx]

**The impact of tunnel mutations on enzymatic catalysis depends on the tunnel-substrate complementarity and the rate-limiting step**

**Piia Kokkonen^1^, Michaela Slanska^1^, Veronika Dockalova^1^, Gaspar P. Pinto^2^, Esther M. Sánchez-Carnerero^3^, Petr Klán^3^, Jiri Damborsky^1,2^, Zbynek Prokop^1,2,*^, David Bednar^1,2,*^**

^1^ Loschmidt Laboratories, Department of Experimental Biology and RECETOX, Faculty of Science, Masaryk University, Brno, Czech Republic

^2^ International Centre for Clinical Research, St. Ann’s Hospital, Brno, Czech Republic

^3^ Department of Chemistry and RECETOX, Faculty of Science, Masaryk University, Brno, Czech Republic

**SUPPORTING INFORMATION**

Contents

[Figures of the Markov states – DBE 2](#_Toc25736502)

[Figures of the Markov states – BDP 3](#_Toc25736503)

[Figures of the Markov states – COU 4](#_Toc25736504)

[The usage of p3 in LinB86-DBE simulations 6](#_Toc25736505)

[Implied timescale plots of the Markov state models 7](#_Toc25736506)

[DBE: LinBwt, LinB32 and LinB86 7](#_Toc25736507)

[BDP: LinBwt, LinB32 and LinB86 7](#_Toc25736508)

[COU: LinBwt, LinBwt with docked COU, LinB32 and LinB86 7](#_Toc25736509)

[Chapman-Kolmogorov tests of the Markov state models 8](#_Toc25736510)

[DBE: LinBwt, LinB32 and LinB86 8](#_Toc25736511)

[BDP: LinBwt, LinB32 and LinB86 9](#_Toc25736512)

[COU: LinBwt, LinBwt with docked COU, LinB32 and LinB86 10](#_Toc25736513)

[Kinetics of LinB32 and LinB86 with BDP. 11](#_Toc25736514)

# Figures of the Markov states – DBE


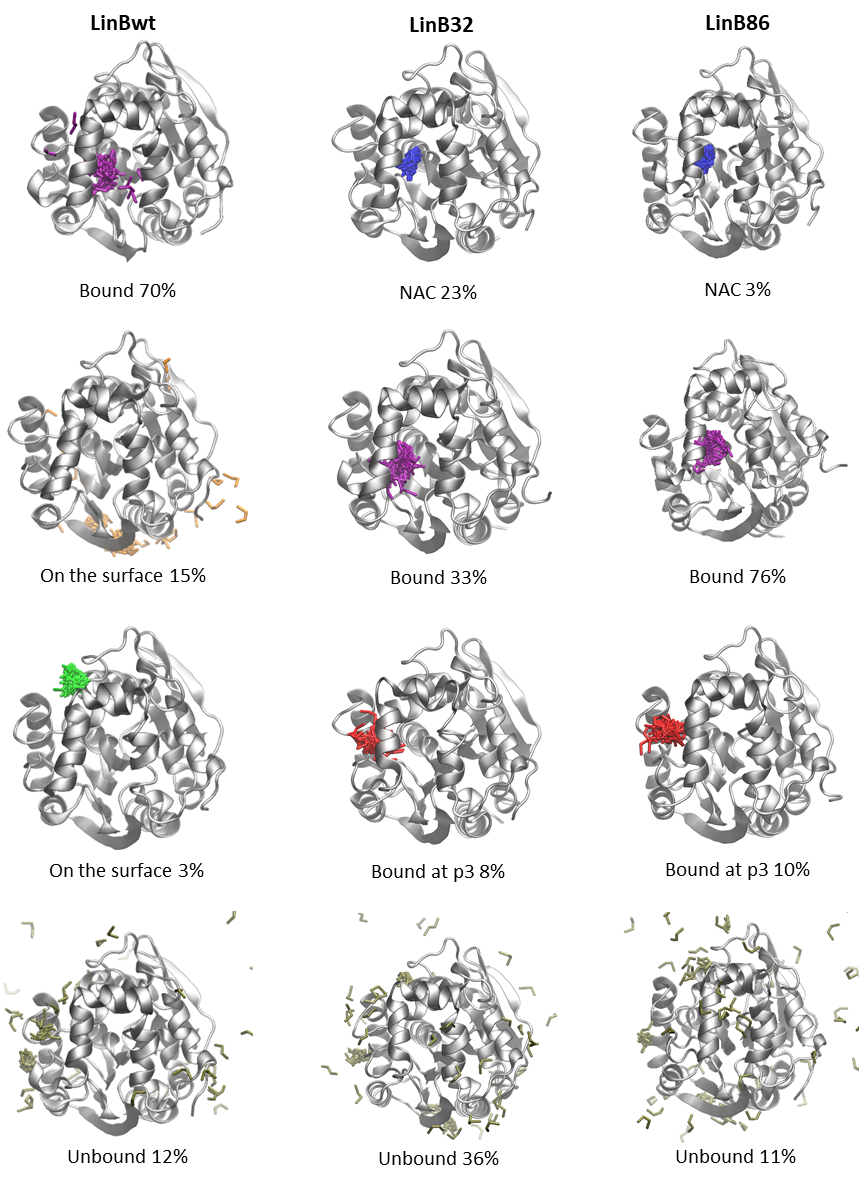


**Figure S1.** The four Markov states observed in DBE simulations.

# Figures of the Markov states – BDP


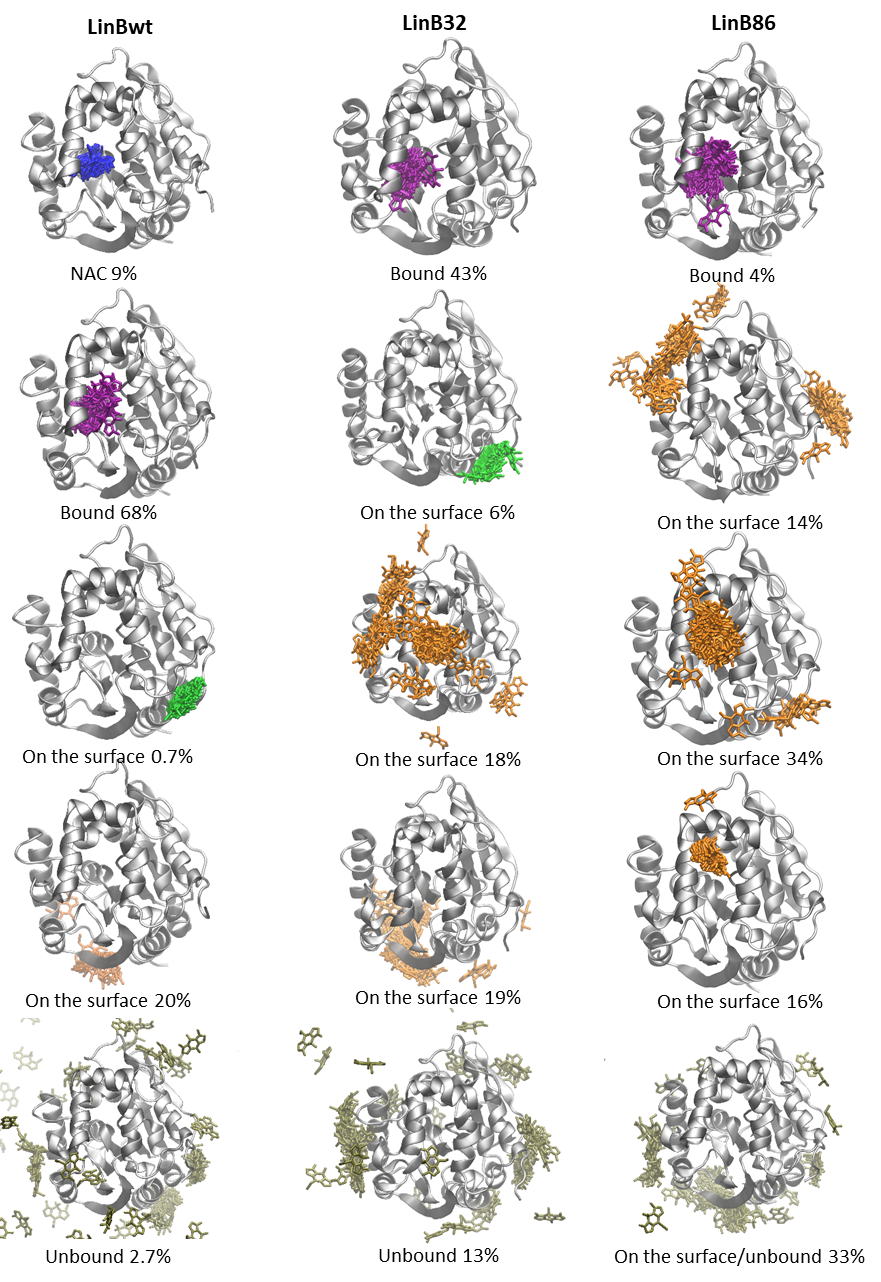


**Figure S2.** The five Markov states observed in BDP simulations.

# Figures of the Markov states – COU


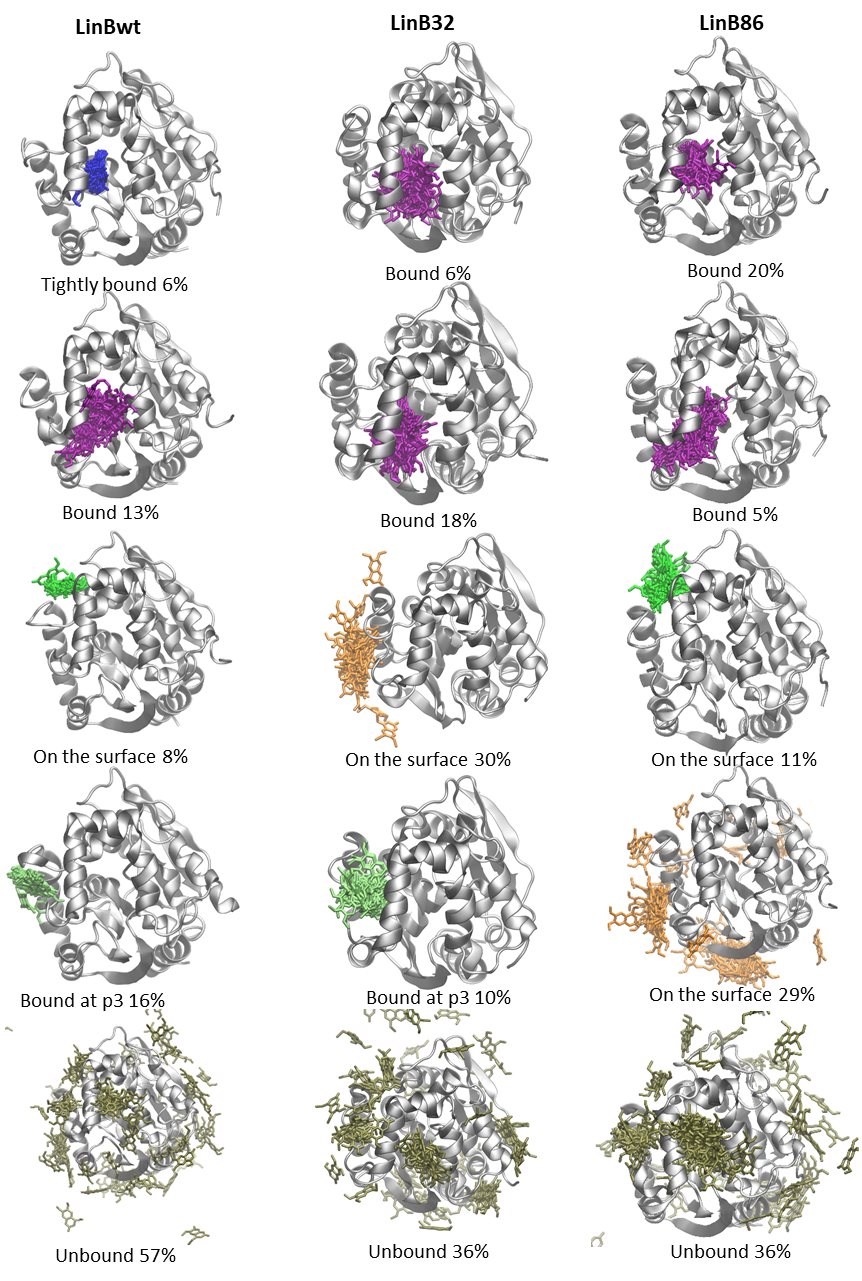


**Figure S3.** The five Markov states observed in BDP simulations.


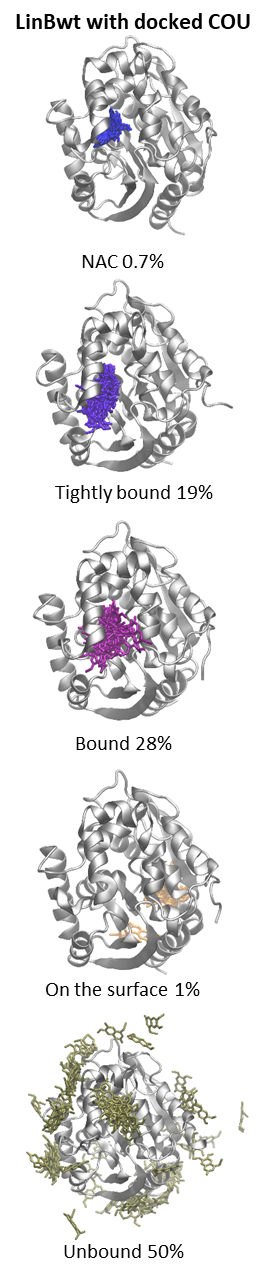


**Figure S4**. The five Markov states observed in simulations where COU was docked to the active site.

# The usage of p3 in LinB86-DBE simulations

**Table S1.** The usage of different binding routes of DBE in LinB86.

| **Path description** | **Usage %** | **Path flux** |
| --- | --- | --- |
| Unbound-bound-NAC | 75.4% | 1.72e-05 |
| Unbound-bound at p3-bound-NAC | 24.6% | 5.61e-06 |
| Unbound-NAC | <0.1% | 9.63e-09 |


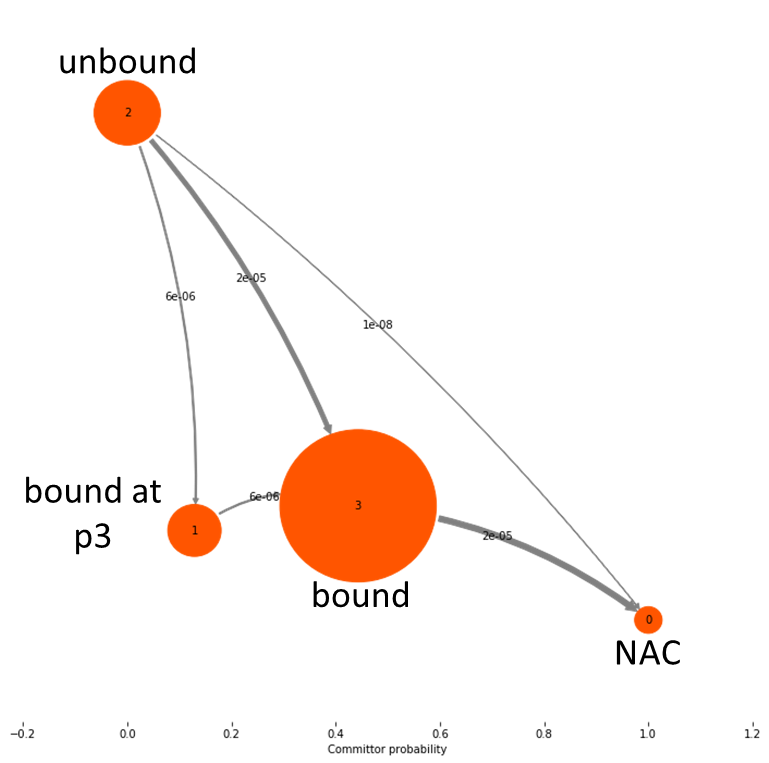


**Figure S5.** The flux pathways for DBE binding in LinB86. The size of the state is relative to its equilibrium probability.

# Implied timescale plots of the Markov state models

## DBE: LinBwt, LinB32 and LinB86


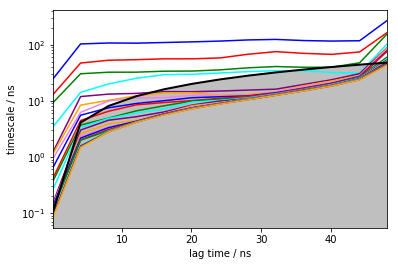

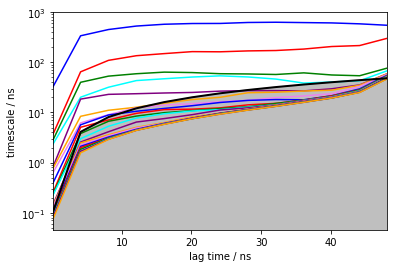

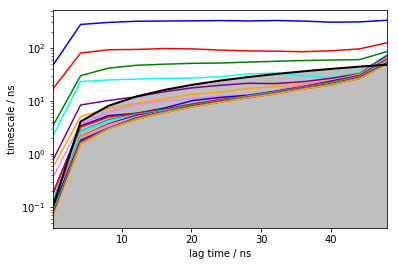


## BDP: LinBwt, LinB32 and LinB86


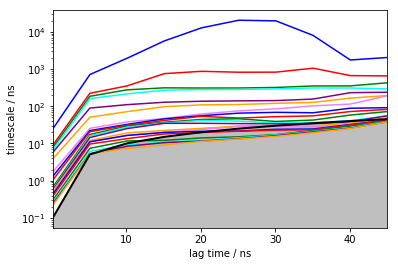

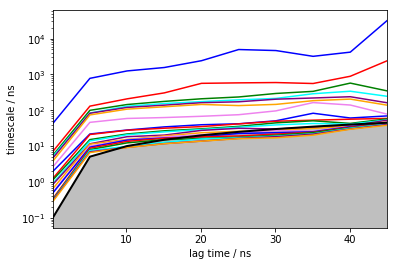

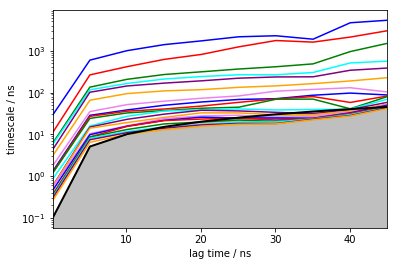


## COU: LinBwt, LinBwt with docked COU, LinB32 and LinB86


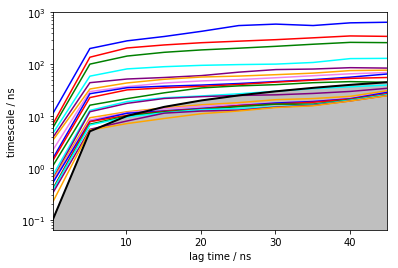

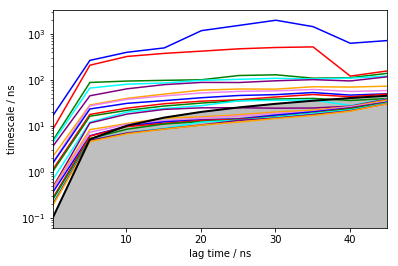

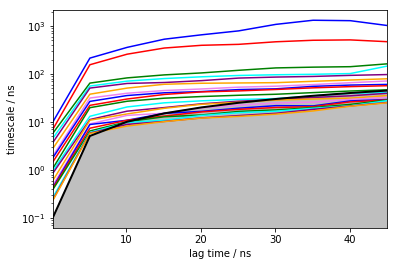

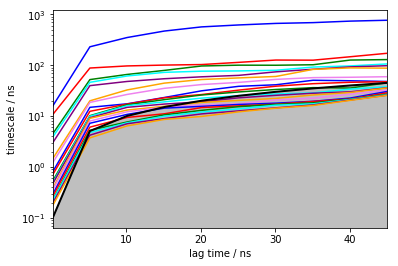


**Figure S6.** Implied time scales plot for all the simulations. The implied time-scale plot is used to estimate if the simulations show Markovian behavior, a suitable lag time for the calculations and an estimate the number of the Markov states. It shows a calculated MSM for each different lag time, showing the different observed states as differently colored lines. It shows that simulations are Markovian (i.e. they are sampling the metric at least reasonably) if the colored lines (individual markov states at different lag times) do not drop significantly when the lag time is increased.

# Chapman-Kolmogorov tests of the Markov state models

## DBE: LinBwt, LinB32 and LinB86


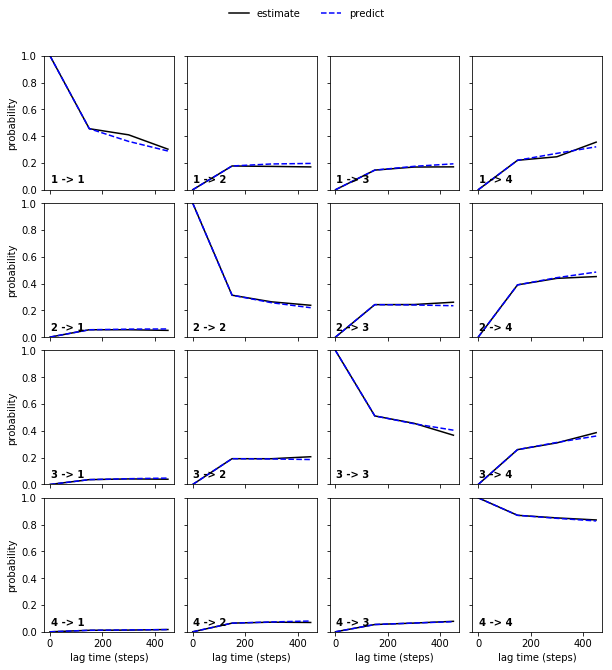

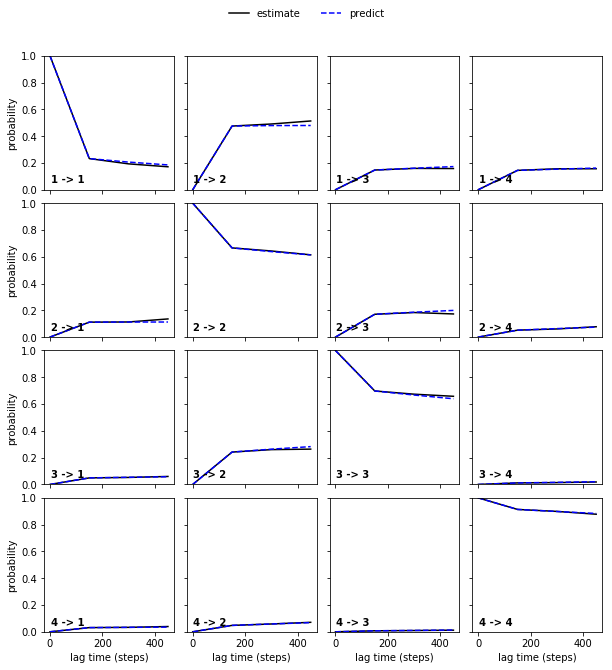


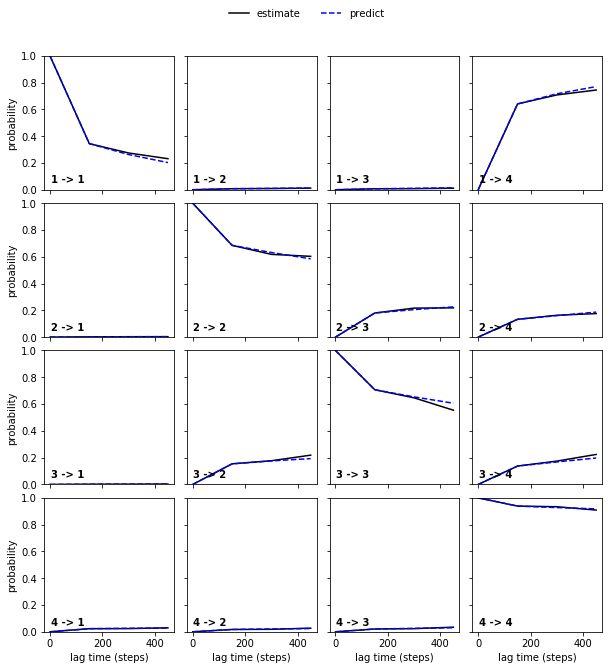


**Figure S7**. Chapman-Kolmogorov tests of the Markov state models for the simulations of DBE. The Chapman-Kolmogorov test is used to test the prediction accuracy of the generated MSM. Basically, it generates a prediction of the simulations based on the model (predict) and compares it with direct estimates from the simulation data (estimate). A well-predictive model will have the two lines overlapping in the plot.

## BDP: LinBwt, LinB32 and LinB86


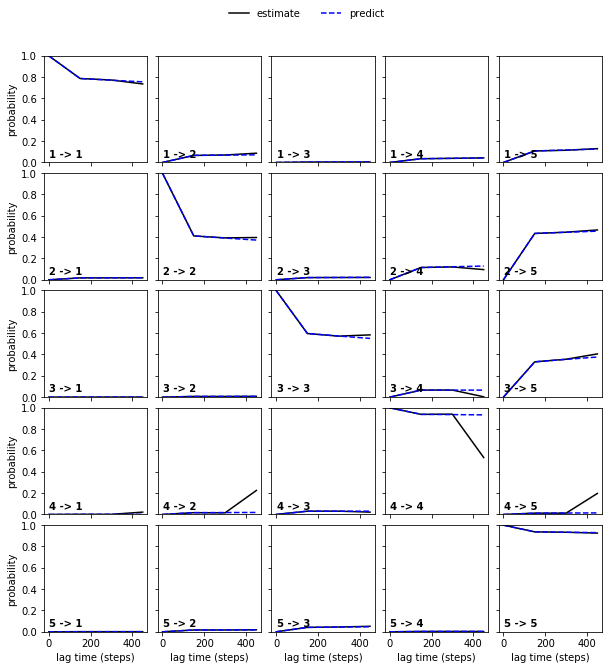

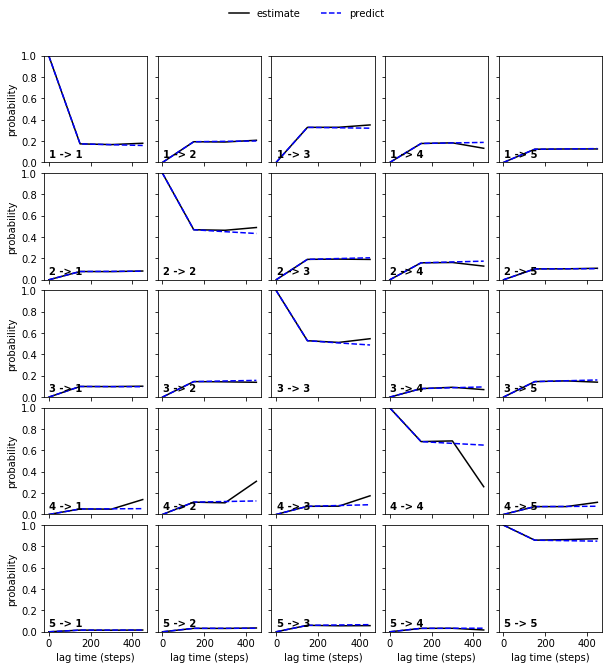


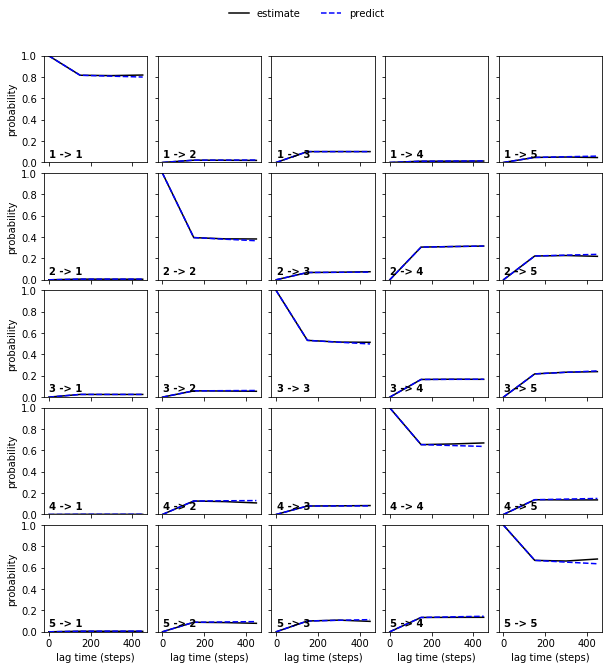


**Figure S8**. Chapman-Kolmogorov tests of the Markov state models for the simulations of BDP. The Chapman-Kolmogorov test is used to test the prediction accuracy of the generated MSM. Basically, it generates a prediction of the simulations based on the model (predict) and compares it with direct estimates from the simulation data (estimate). A well-predictive model will have the two lines overlapping in the plot.

## COU: LinBwt, LinBwt with docked COU, LinB32 and LinB86


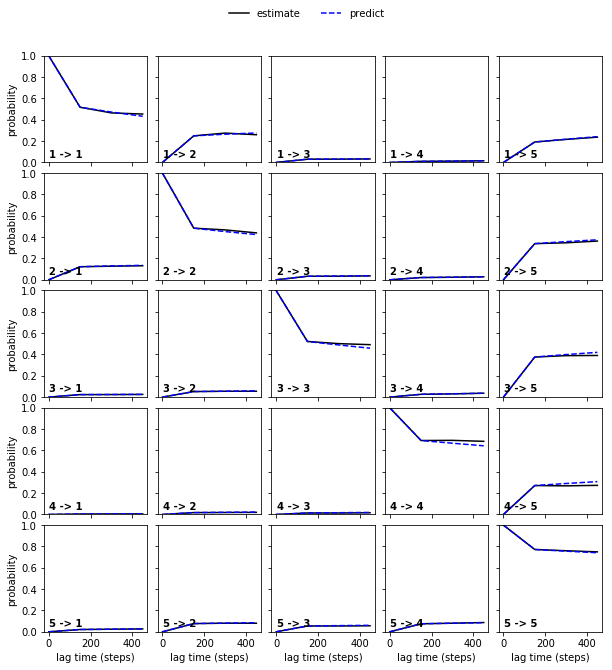

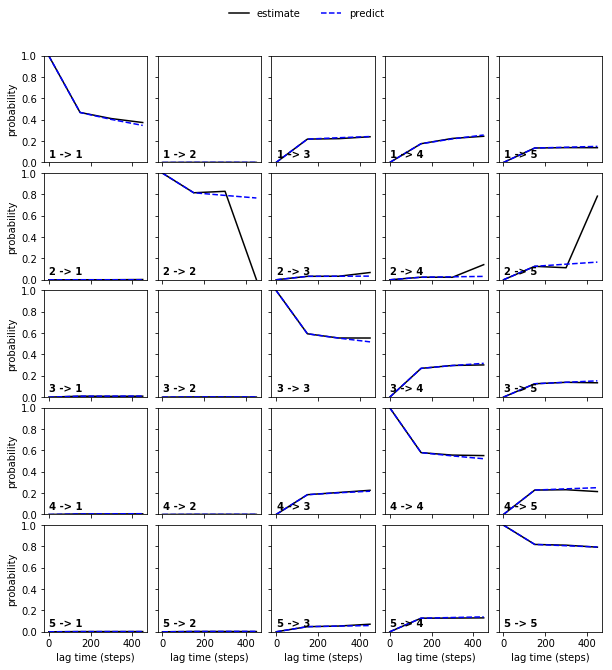


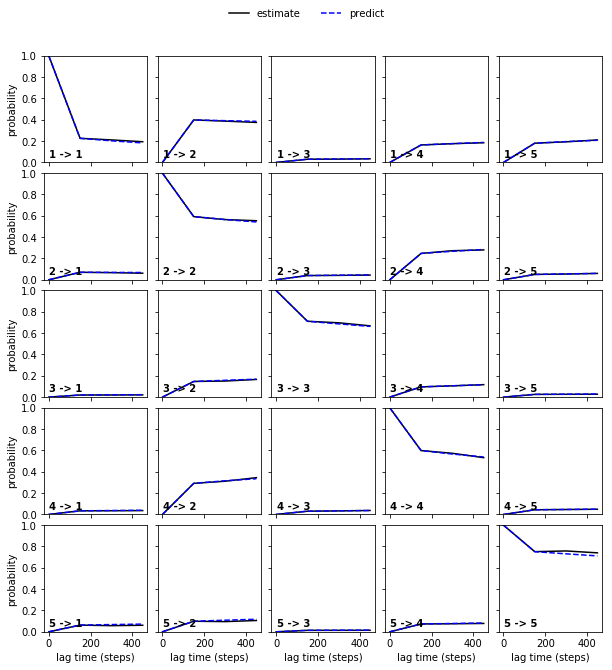

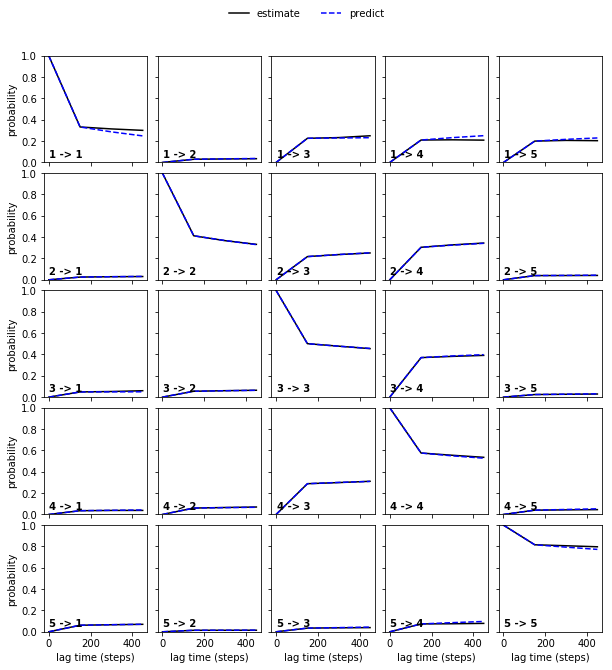


**Figure S9**. Chapman-Kolmogorov tests of the Markov state models for the simulations of COU. The Chapman-Kolmogorov test is used to test the prediction accuracy of the generated MSM. Basically, it generates a prediction of the simulations based on the model (predict) and compares it with direct estimates from the simulation data (estimate). A well-predictive model will have the two lines overlapping in the plot.

# Kinetics of LinB32 and LinB86 with BDP

**A B**

**
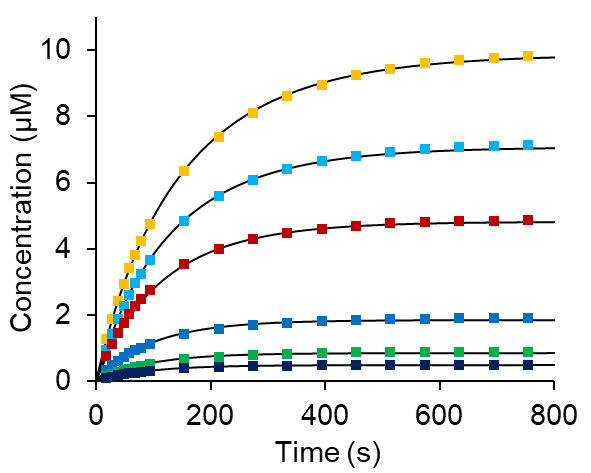

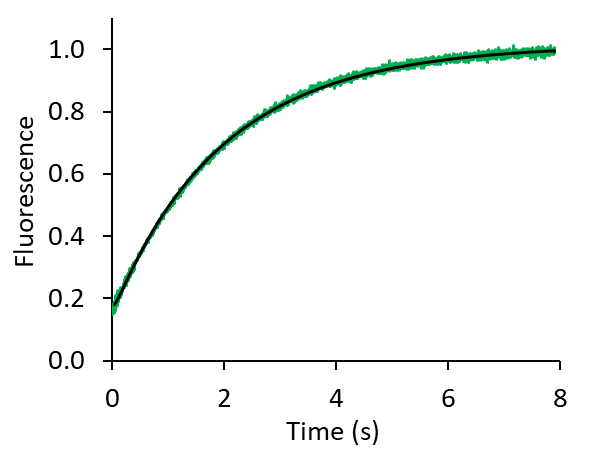
**

**C D**

**
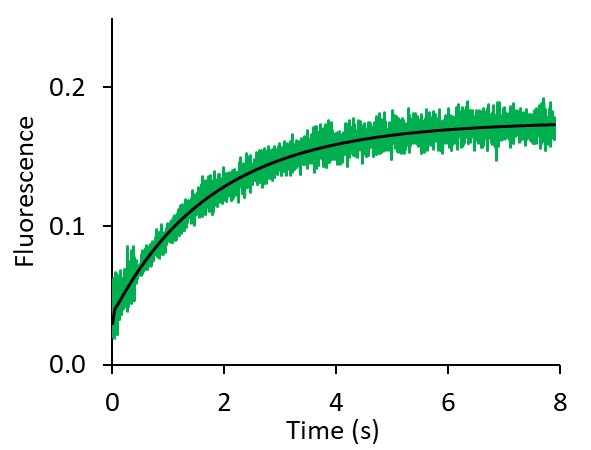

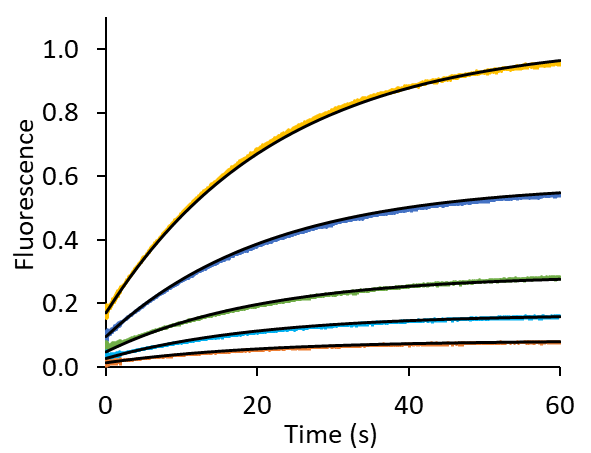
**

**E**

**
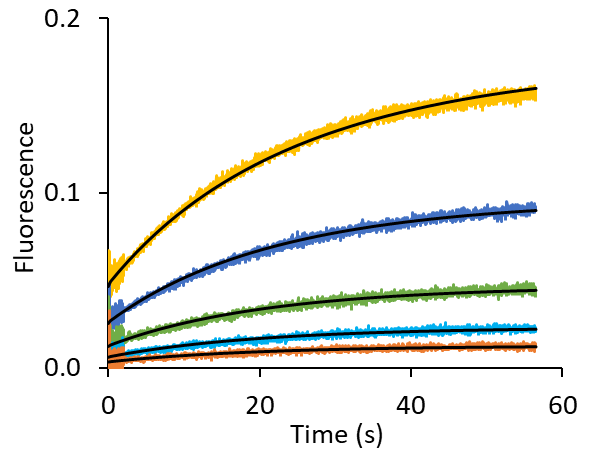
**

**Figure S10**. K**inetics of BDP conversion by LinB32.** Experiments were performed at 30°C in phosphate buffer pH 8.0 with 10% DMSO. Steady-state data (A) obtained by full conversion of 0.5, 1, 2, 5, 7 and 10 μM BDP by 0.14 μM LinB32. Single turnover experiments were carried out with the excitation wavelength of 500 nm (B) for specific excitation of the fluorogenic substrate and 280 nm (C) for specific excitation of tryptophan residues utilized as a FRET donor to transfer the energy to bound BDP. The concentration of LinB32 and BDP was 10 μM and 5 μM, respectively. The concentration dependence was analyzed by mixing 0.65 μM enzyme with different concentrations of BDP (0.3, 0.6, 1.25, 2.5 and 5 μM). These data were recorded for the excitation wavelength 500 nm (D) and 280 nm (E). Each trace represents an average of six to ten repetitions. The black lines represent the best global fit. Scaling factors of the fluorescence signal were included in global fit as parameters (*a* = 4.3±0.1; *b* = 6.2±0.1; *o* = 1.8±0.5; *p* = 4.7±2.7; *q* = 7.4±0.8 and *r* = 3.6±0.1).

**A B**

**
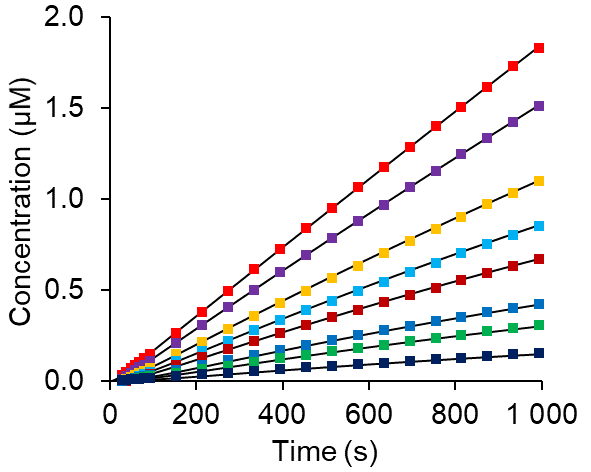

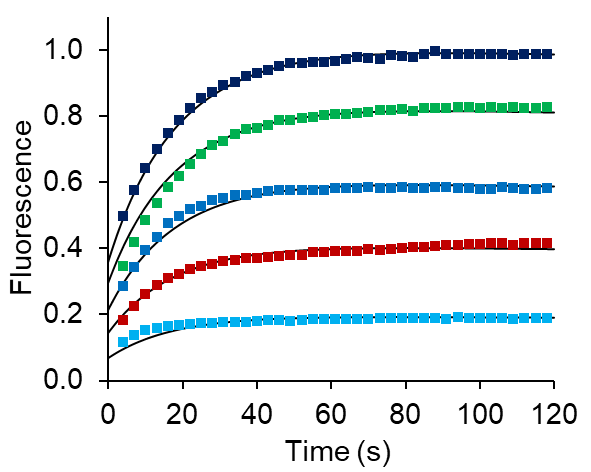
**

**C D**

**
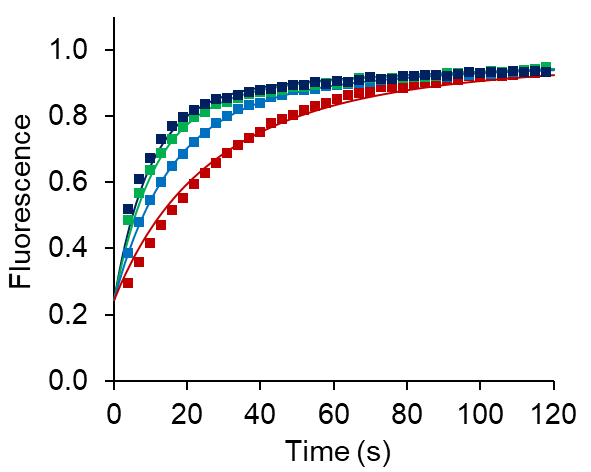

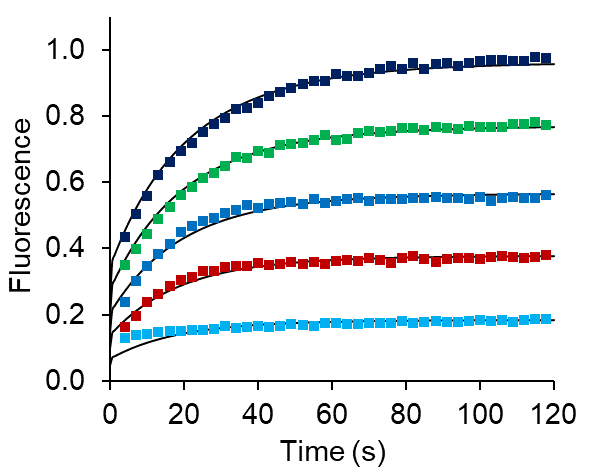
**

**Figure S11**. **Kinetics of BDP conversion by LinB86.** Experiments were performed at 30°C in phosphate buffer pH 8.0 with 10% DMSO. Steady-state data (A) obtained by recording initial phases of conversion of 1, 2, 3, 5, 7, 10, 15 and 20 μM BDP by 0.2 μM LinB86. The concentration dependence of the single-turnover kinetics was analyzed for the excitation wavelength 500 nm at fixed enzyme concentration of 30 μM with different concentrations of BDP 2, 4, 6, 8 and 10 μM (B) and at fixed concentrations of BDP (10 μM) and variable concentration of enzyme 20, 30, 40 and 50 μM (C). The substrate concentration dependence 2, 4, 6, 8 and 10 μM BDP was analysed at 280 nm (D) for specific excitation of tryptophan residues utilized as a FRET donor to transfer the energy to bound BDP. The concentration of LinB86 was 30 μM. Each trace represents an average of six to ten repetitions. The black lines represent the best global fit. Scaling factors of the fluorescence signal were included in global fit as parameters (*a* = 3.4±0.4; *b* = 3.9±0.5; *o* = 2.3±0.1; *p* = 3.6±0.2; *q* = 3.8±0.2 and *r* = 4.0±0.5).

**
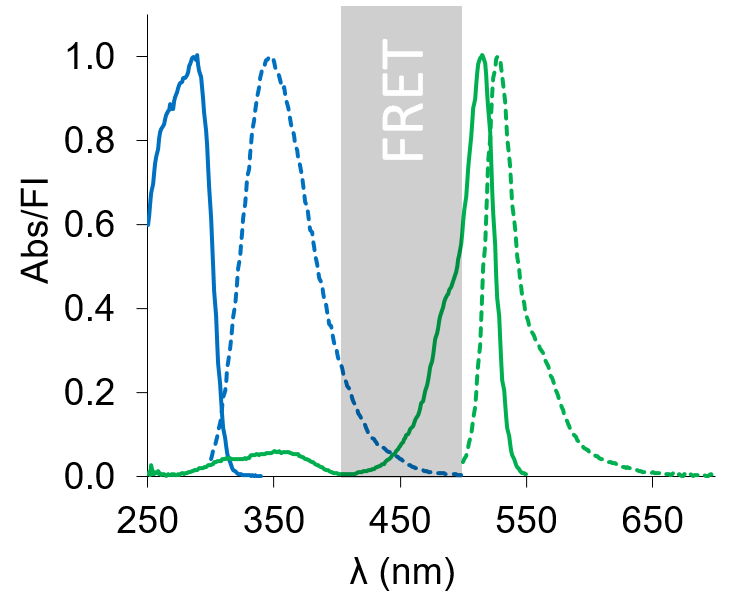
**

**Figure S12**. **Normalized absorption and emission spectra of BDP and tryptophan.** Absorption (solid lines) and emission (dashed lines) spectra of tryptophan/NATA (blue) and BDP (green). The spectra were recorded under reaction conditions, PBS buffer/DMSO mixture (90:10, v/v), pH 8.0. The grey zone indicates the region where tryptophan as a FRET donor transfer the energy to BDP.

**Table S1**. **Error analysis on fitted kinetic parameters.** The best ﬁt was derived by nonlinear regression based on numerical integration of the rate equations as described in the text. The lower and upper limits for each parameter were derived from the confidence contours for χ^2^ threshold at boundary 0.98

| LinB32 | | | | LinB86 | | | |
| --- | --- | --- | --- | --- | --- | --- | --- |
| parameter | best-fit | lower | upper | parameter | best-fit | lower | upper |
| *k*_+1_ | 2.8 | 1.89 | 5.68 | *k*_+1_ | 0.0244 | 0.0137 | 0.363 |
| *k*_-1_ | 65 | 45 | 134 | *k*_-1_ | 1.7 | 1.25 | 71.1 |
| *k*_+2_ | 2.1 | 1.99 | 2.13 | *k*_+2_ | 0.184 | 0.165 | 0.532 |
| *k*_3_ | 12 | 10.0 | 19.5 | *k*_3_ | 0.0109 | 0.0106 | 0.0111 |
| *k*_4_ | - *^a^* | - *^a^* | - *^a^* | *k*_4_ | 17.9 | 13.2 | 19.3 |
| *k*_-4_ | 11.3 | 10.6 | 11.7 | *k*_-4_ | 4.27 | 3.99 | 5.76 |
| *f1* | 0.222 | 0.221 | 0.223 | *f1* | 0.323 | 0.28 | 0.328 |
| *a* | 4.27 | - *^b^* | 4.34 | *a* | 3.43 | 3.40 | 4.07 |
| *b* | 6.18 | 6.09 | 6.24 | *b* | 3.92 | 3.87 | 5.13 |
| *f2* | 0.00974 | 0.00974 | 0.00979 | *f2* | 0.0281 | 0.0224 | 0.0286 |
| *o* | 1.83 | 1.81 | 2.06 | *o* | 2.33 | 2.28 | 2.34 |
| *p* | 4.72 | - *^b^* | 5.15 | *p* | 3.58 | 3.43 | 3.60 |
| *q* | 7.41 | 7.37 | 7.54 | *q* | 3.84 | 3.84 | 4.08 |
| *r* | 3.59 | 3.57 | 3.59 | *r* | 4.06 | 4.05 | 4.15 |
| *^a^* fixed value, not used as fitted parameter | | | |  |  |  |  |
| *^b^* no lower limits on the parameter obtained | | | |  |  |  |  |
